# Assessing the effectiveness of embalming in cadavers with COVID-19 after full autopsy and minimally invasive autopsy

**DOI:** 10.1007/s12024-026-01225-6

**Published:** 2026-04-10

**Authors:** Hisako Saitoh, Sayaka Nagasawa, Aika Mori, Yuichiro Hirata, Harutaka Katano, Kazuho Ushiwata, Suguru Torimitsu, Makoto Nakajima, Shigeki Tsuneya, Susumu Kobayashi, Isao Yamamoto, Kimiko Nakagawa, Iwao Hasegawa, Shinji Akitomi, Koichi Sakurada, Tadaki Suzuki, Hirotaro Iwase, Kenichiro Hashizume, Yohsuke Makino

**Affiliations:** 1 Department of Forensic Dentistry, Graduate School of Medicine and Dental Sciences, Institute of Science Tokyo, 1-5-45 Yushima, Bunkyo-Ku, Tokyo 113-8510 Japan; 2 Department of Legal Medicine, Graduate School of Medicine, Chiba University, Chiba, Japan; 3 Laboratory of Forensic Toxicology, Graduate School of Pharmaceutical Sciences, Chiba University, Chiba, Japan; 4 Department of Infectious Disease Pathology, National Institute of Infectious Diseases, Japan Institute for Health Security, Tokyo, Japan; 5 GSI CO., Ltd., Tokyo, Japan; 6 Department of Forensic Medicine, Graduate School of Medicine, The University of Tokyo, Tokyo, Japan; 7 Department of Forensic Medicine, Kanagawa Dental University, Kanagawa, Japan; 8 Public Interest Incorporated Association, Nihon Kousei-Kyoukai, Kanagawa, Japan; 9 Japan Medical Association Research Institute, Tokyo, Japan; 10 International Research Institute of Disaster Science, Tohoku University, Miyagi, Japan; 11 Kanazawa Medical University, Emergency Medicine, Ishikawa, Japan; 12 General Incorporated Association Greif Support Institute, Tokyo, Japan

**Keywords:** Minimally invasive autopsy, Autopsy, Embalming, COVID-19, SARS-CoV-2, Powered air-purifying repirators

## Abstract

Embalming (EM) reduces residual SARS-CoV-2 in tissues, but its effectiveness on the external body surfaces of corpses infected with coronavirus disease 2019(COVID-19) remains unclear. We therefore examined the presence of SARS-CoV-2 in the pharynx, anus, and on external body surfaces following EM in nine COVID-19-infected corpses, including four full autopsy cases and five minimally invasive autopsy (MIA) cases. Antigen testing and viral quantification using real-time PCR were performed. Nasopharyngeal antigen tests were positive prior to EM and negative thereafter in all nine cases. Among the four autopsy cases, SARS-CoV-2 RNA was detected in all nasopharyngeal samples (100%), in two oropharyngeal samples (50%), and at seven sites (41%) across 17 external body surfaces, three or more days after EM. No viral RNA was detected in anal samples. Among the five MIA cases, viral RNA was detected in two nasopharyngeal samples (40%), one oropharyngeal sample (20%), and at four sites (44%) across nine external body surfaces, three or more days after EM. The reduction in viral load before and after EM exceeded 99%in nasopharynx and oropharyngeal samples in both autopsy and MIA cases, and exceeded 94% on external body surfaces in MIA cases. Moreover, viral reduction was greater several days after EM than immediately following the procedure. These findings suggest that the combination of MIA and EM effectively reduces the detectableSARS-CoV-2 RNA in COVID-19-infected corpses. Additionally, maintaining the body for several days after EM appears to reduce the viral levels.

**Supplementary Information:**

The online version contains supplementary material available at https://doi.org/10.1007/s12024-026-01225-6.

## Key points

In autopsy and MIA cases, nasopharyngeal antigen tests were positive before EM but negative after EM.

In four autopsy cases, SARS-CoV-2 was detected in the nasopharynx in all (100%) and in the oropharynx in two cases (50%) after EM. Among the 17 external surface sites, the virus was detected in six (35%) 1 d and in seven (41%) 3 d after EM. Anal swabs were negative before and after EM in all cases.

In five MIA cases, SARS-CoV-2 was detected in the nasopharynx of three (60%) immediately after EM and in two (40%) 3 d thereafter. In the oropharynx, the virus was detected in one case (20%) both immediately and 3 d after EM. In nine external surface sites, the virus was detected in one (11%) immediately after EM and in four sites (44%) 3 d thereafter

The amount of SARS-CoV-2 decreased by 99% in the nasopharynx and oropharynx and by 94% on external surfaces after EM. In many cases, EM appeared more effective several days after treatment.

**Supplementary Information:**

The online version contains supplementary material available at https://doi.org/10.1007/s12024-026-01225-6.

## Introduction

From its emergence in December 2019 until the World Health Organization (WHO) declared the end of the public health emergency in May 2023, severe acute respiratory syndrome coronavirus 2 (SARS-CoV-2) caused a global disease outbreak. Numerous studies have examined whether coronavirus disease 2019 (COVID-19) can be transmitted from corpses [1–7]. In May 2021, Plenzig et al. reported infectious SARS-CoV-2 in the lungs of deceased individuals with a maximum postmortem interval of 17 d [3]. In July of that year, they detected viral RNA using PCR in a corpse that had been buried for 4 months [4]. In February 2023, the viral infectivity reached 2.09 × 10^6^ plaque-forming units/g, indicating substantial postmortem viral activity [6]. Transmission from infected carcasses to uninfected hamsters has been demonstrated [7]. Therefore, SARS-CoV-2 may remain highly infectious even after death and corpses/organs may be sources of infection. This raises concerns regarding the infection risks of healthcare workers, coroners, police officers, undertakers, and other professionals occasionally handling cadavers, as well as in the use of cadavers in medical education and research [8, 9].

Much attention has been focused on embalming (EM), a procedure that replaces blood and bodily fluids with formalin-based solutions injected into the vascular system, to preserve and disinfect cadavers for long-term storage. Animal studies have shown that cavity sealing or EM can prevent transmission from cadavers [7]. The effectiveness of EM has also been demonstrated in virus-positive human cases [10–12]. In 2022, Vymazalová et al. showed that the stability of SARS-CoV-2 RNA in corpses treated with fixatives decreased significantly, enabling safer handling, although insufficient perfusion could compromise effectiveness [9]. In 2023, Gitto et al. reported that EM reduced the viral load in tissues, sometimes below the detection limit, but viral RNA persisted [12]. However, detailed data on residual virus particles in the nasopharynx, oropharynx, and external body surfaces before and after EM remain limited.

During the COVID-19 pandemic, when conventional autopsies were restricted owing to biosafety concerns, minimally invasive autopsy (MIA) emerged as an alternative for evaluating the causes of death and disease mechanisms. MIA uses biopsy needles to collect tissue samples for pathological diagnosis and is particularly useful in resource-limited settings [13, 14]. Recent studies confirmed the clinical value, safety, and diagnostic accuracy of MIA in both in-hospital and out-of-hospital SARS-CoV-2-related cases [15–18]. After MIA, EM solution can typically be injected into a single artery and perfused throughout the body. In contrast, post-autopsy cadavers often have their arteries, veins, and organs removed, requiring localized perfusion, subcutaneous injection, or packing, which may reduce perfusion efficiency. Despite these procedural differences, no comparative data on the effectiveness of EM following autopsy and MIA have been reported.

This study addressed two questions: Can EM reduce the number of virus particles remaining on the external surfaces of cadavers with confirmed SARS-CoV-2 infection? Does the effectiveness of EM differ between autopsy and MIA cases? We investigated the SARS-CoV-2 viral load in swab samples from the nasopharynx, oropharynx, anus, and body surfaces before and after EM in autopsy and MIA cases, and evaluated changes over time.

## Materials and methods

### Sample collection

#### Target cases

The study included four COVID-19 cadavers subjected to full autopsy at the Department of Forensic Medicine, Graduate School of Medicine, the University of Tokyo, between August 2021 and July 2022, and five COVID-19 cadavers subjected to MIA at the Department of Forensic Medicine, Kanagawa Dental University, between April and August 2022. Table 1 presents the age, sex, cause of death, time from the first positive PCR to death, and postmortem interval.

**Table 1:** Clinical characteristics and postmortem intervals of autopsy and MIA cases

Group	Case number	Age	Sex	Cause of death	Death–autopsy/MIA interval (days)	Fisrt positive PCR- to-death interval (days)
Autopsy	A1	80 s	F	COVID-19 pneumonia	4	8
	A2	70 s	M	COVID-19 pneumonia	13	0*
	A3	80 s	F	COVID-19 pneumonia, aspiration pneumonia, invasive pulmonary aspergillosis	5	29
	A4	80 s	M	COVID-19 pneumonia	14	1
MIA	M1	90 s	F	Hypoxic encephalopathy due to food aspiration	3	1
	M2	50 s	M	COVID-19 pneumonia	2	2
	M3	70 s	F	Aortic dissection	14	6
	M4	80 s	M	COVID-19 pneumonia	5	2
	M5	50 s	M	COVID-19 pneumonia	4	2

^*^ PCR positivity confirmed after death

#### EM and specimen collection

**Fig. 1:**
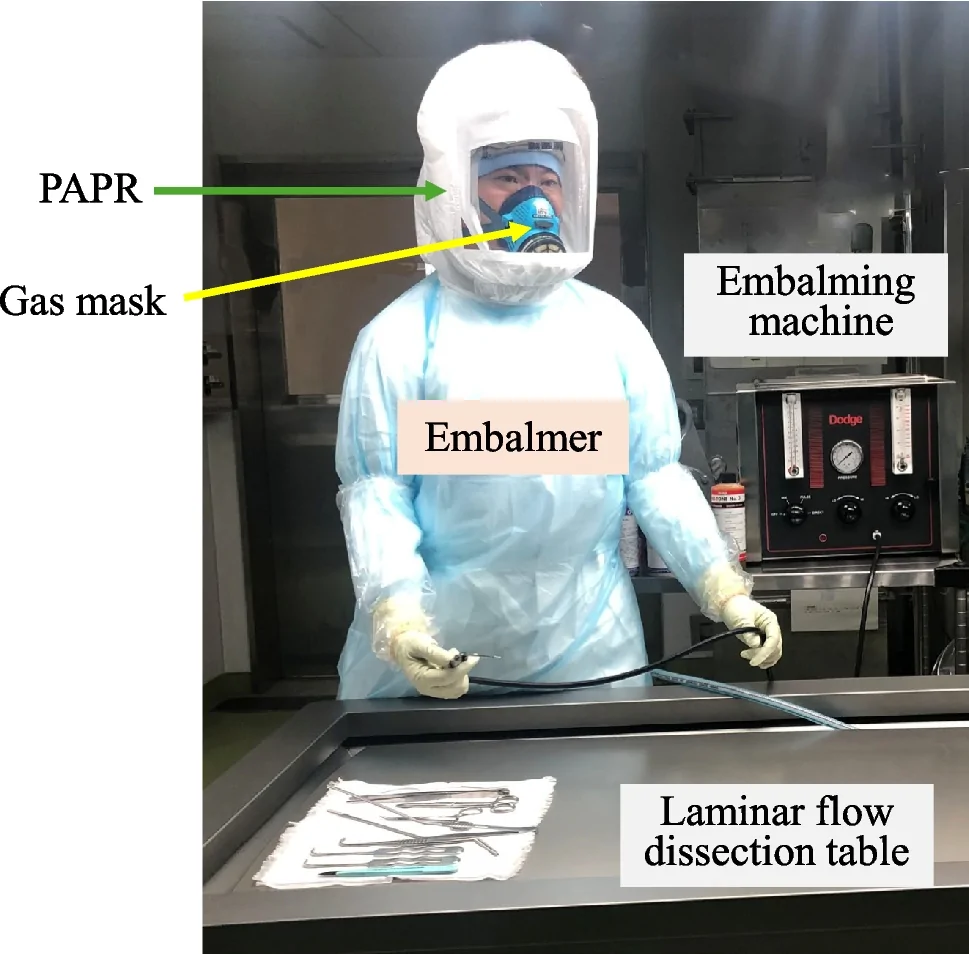
Infection control measures for an embalmer during EM of a cadaver infected with COVID-19. The embalmer wore a gas mask and powered air-purifying respirator (PAPR) protecting against formaldehyde gas and COVID-19, and performed the embalming on a laminar flow dissection table. As of August 2021 to July 2022, PAPRs that can protect against both formaldehyde gas and virus particles were not available for sale in Japan

Although the WHO guidelines (September 2020) do not recommend EM because of manipulation risks, nonetheless, it should be conducted by trained staff under standard IPC precautions [19, 20]. All procedures were conducted in an autopsy room compliant with the Centers for Disease Control and Prevention guidelines (ventilation rate ≥ 12 air changes per hour and a laminar flow autopsy table) [21]. The personnel wore full personal protective equipment, including powered air-purifying respirators (Versaflow 3 M TR-300 + HKL; 3 M Japan Ltd., Tokyo, Japan; Fig. 1). After examination, the bodies were placed in new body bags over disposable garments and stored in a refrigerator. All sampling was performed on a laminar flow dissection table by two operators wearing personal protective equipment (PPE). All work was conducted under strict infection control measures, and no staff members were infected with SARS-CoV-2.

#### EM

##### Autopsy cases

###### **Cleaning and disinfection of corpses**

Following dissection, EM was initiated without suturing. The body surface, hair, and skin were washed with detergent to remove residual blood and tissue debris. The eyes, nasal, and oral cavities were wiped with cotton soaked in 60% isopropyl alcohol. A gel-like EM agent (10% formaldehyde-containing gel) was applied to the nasal cavity, nasopharynx, oral cavity, oropharynx, anus, body cavities, cranial cavity, and isolated sternum.

###### **Perfusion of EM agent, subcutaneous injection, or reapplication of gel-type EM agents**

Formaldehyde solution (5–10% formaldehyde solution) was perfused into the bilateral common iliac, subclavian, and facial arteries using an EM machine (Dodge Embalming Machine EM524 240 V UK; Dodge Co., Billerica, MA, USA), with blood drained through the corresponding veins. When perfusion was not possible or insufficient, the agent was injected subcutaneously. Blood in the cranial and body cavities was suctioned and wiped away, and gel-based EM agent (10% formaldehyde-containing gel) was reapplied in the cranial and body cavities.

###### **Post-perfusion treatment and washing**

Along with the intact gel-type EM agent in the cranial and body cavities, and these cavities were filled with a high-molecular-weight absorbent material. Incisions were sutured and residual surface contaminants were washed off.

##### MIA cases

###### **Cleaning and disinfection of corpses**

After MIA, the entire body surface was washed with detergent and the eyes, nasal cavity, and oral cavity were wiped with cotton soaked in 60% isopropyl alcohol. The nasal and oral cavities were filled with cotton soaked in EM agent (5–7% formaldehyde solution).

###### **Perfusion of EM agent and subcutaneous injection**

Small incisions (1.5–2.0 cm) were made in the upper right clavicle and right or left thigh, and the carotid and iliac arteries were perfused with 5–7% formaldehyde solution using an EM machine. Blood was drained from the corresponding veins. A trocar was used to instill a 21% formaldehyde solution into the thoracic and abdominal cavities.

###### **Post-perfusion treatment and washing**

All incisions were sutured, and the body surface was washed.

#### Target specimens

For autopsy cases, nasopharyngeal, oropharyngeal, and anal swabs were collected before dissection. After EM, swab samples were collected from 17 external body sites, including the head, face, neck, hands, and feet, on days 3, 4, and 7.

For MIA cases, nasopharyngeal, oropharyngeal, and nine external surface swabs were collected before MIA, immediately after EM and on days 3, 4, or 5.

Nasopharyngeal, oropharyngeal, and anal swab samples were immersed in virus transport fluid (SUGIYAMA-GEN Co., Ltd., Tokyo, Japan), and external surface swab samples were placed in 15-mL sterile tubes and stored at − 80 °C.

### Antigen test

Nasopharyngeal swab samples were tested using a SARS-CoV-2 antigen qualitative kit (QuickNavi™-COVID19 Ag; Otsuka Pharmaceutical Co., Ltd., Tokyo, Japan) [22].

### RNA extraction

Nucleic acids were extracted from nasopharyngeal swabs collected before autopsy using a Maxwell RSC Viral Total Nucleic Acid Kit (Promega, Madison, WI, USA). For other specimens, nucleic acid was extracted using an EZ1^®^ Virus Mini kit v2.0 (QIAGEN GmbH, Hilden, Germany) and EZ1 Advanced XL system (QIAGEN).

### Virus quantification using real-time PCR

In nasopharyngeal swabs collected before autopsy, 1 µL of nucleic acid solution was used to detect and quantify SARS-CoV-2 RNA using RT-qPCR [18]. In other samples, the following reagents were used: Primer/Probe Set (2019-nCoV; Takara Bio Inc., Shiga, Japan), One Step PrimeScript™ Ⅲ RT-qPCR Mix with UNG (Takara), and Positive Control RNA Mix (2019-nCoV; Takara). The 20 µL reaction mixture contained 10.0 µL One Step Prime Script, 4.0 µL Primer/Probe mix for the N1 and N2 regions, 0.4 µL Rox Reference Dye, 0.6 µL RNase free H_2_O, and 5.0 µL of extracted RNA as template. Real-time PCR was performed using a StepOnePlus™ Real-Time PCR System (Thermo Fisher Scientific, Waltham, MA, USA). The reaction conditions included UNG treatment at 25 °C for 10 min, reverse transcription at 52 °C for 5 min and 95 °C for 5 s, followed by 40 cycles of 95 °C for 5 s and 60 °C for 30 s. If either the N1 or N2 region demonstrated < 40 cycles, the sample was considered PCR-positive, and the viral load was measured.

### Statistical analysis

A two-tailed Fisher's exact test was performed to assess differences in virus detection in the nasopharynx, oropharynx, and external body surface at three time points (immediately after EM, the next day, and ≥ 3 d after EM) between autopsy and MIA cases, with significance set at *p* < 0.05.

## Results

### Antigen test results using nasopharyngeal swabs

In all nine cases, antigen tests using nasopharyngeal swabs were positive before autopsy or MIA and negative after EM.

### Real-time PCR results for nasopharynx, oropharynx, and anus before and after EM in autopsy cases

In postmortem swab samples, late-cycle amplification may increase nonspecific signals due to RNA degradation and postmortem changes; therefore, amplification was limited to 40 cycles to minimize false-positive results and ensure reliable detection.

**Fig. 2:**
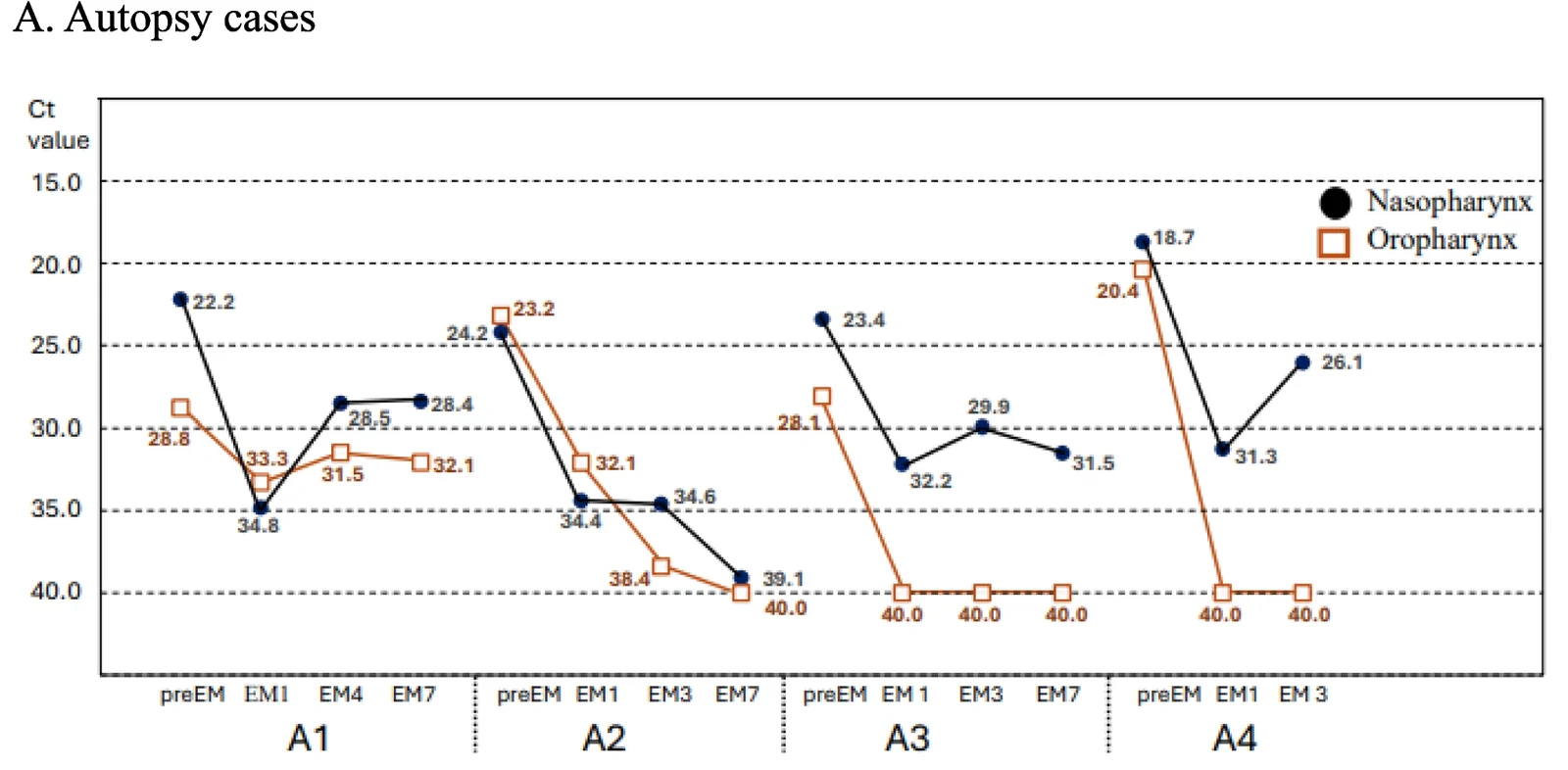

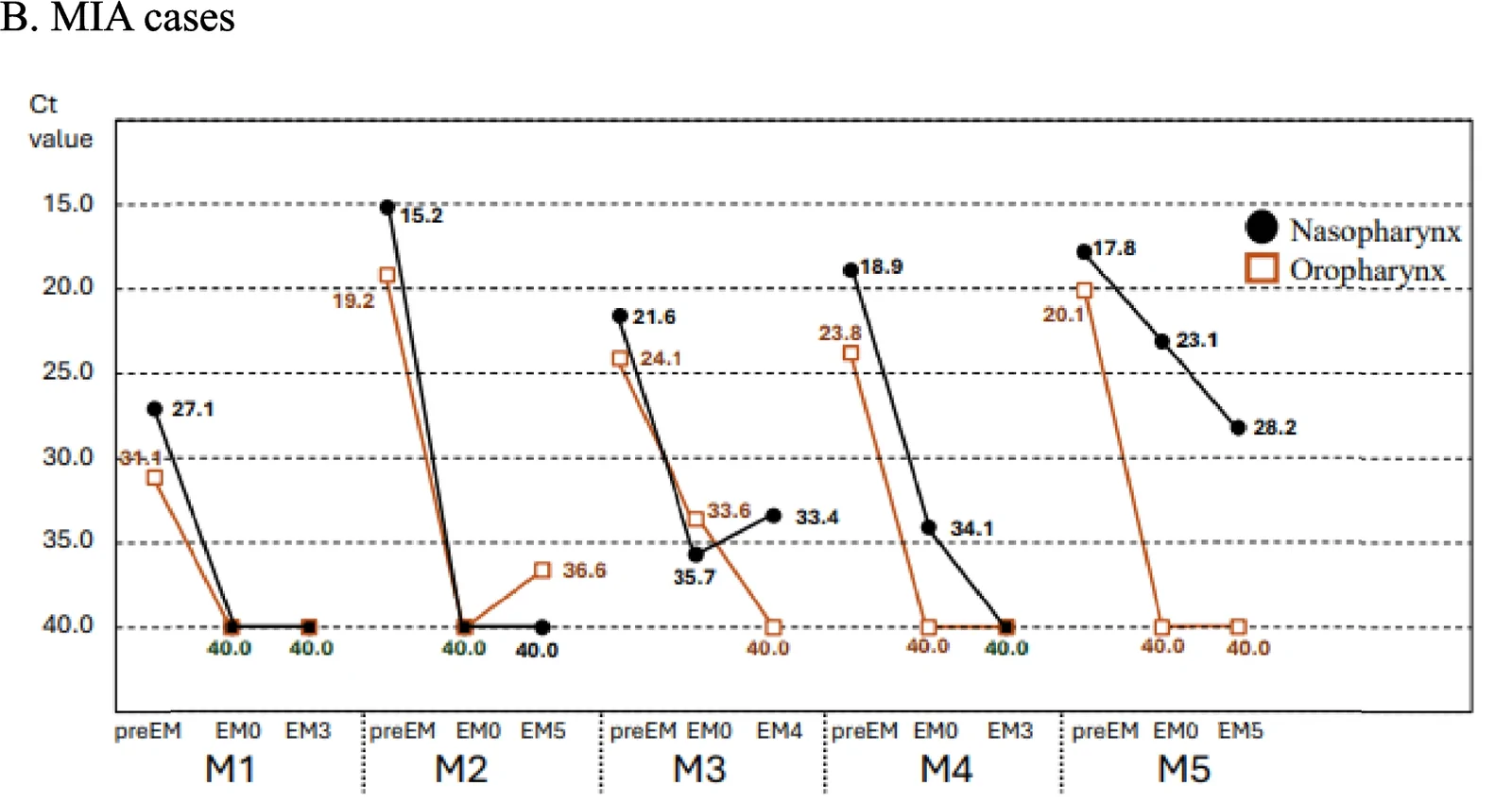
Real-time PCR results for SARS-CoV-2 (N2 region) before and after EM in cadavers infected with COVID-19. **A** Autopsy cases. The vertical axis shows Ct values, with black circles indicating nasopharyngeal measurements and orange squares indicating oropharyngeal measurements. The horizontal axis shows the time elapsed after EM for four autopsies (A1, A2, A3, and A4). “pre EM” indicates the time before dissection and EM; “EM1” indicates the day after EM; “EM3” indicates the third day after EM; “EM4” indicates the fourth day after EM; and “EM7” indicates the seventh day after EM, representing the specimen collection date. **B** MIA cases. The vertical axis shows Ct values, with black circles indicating nasopharyngeal measurements and orange squares indicating oropharyngeal measurements. The horizontal axis indicates the MIA5 cases (M1, M2, M3, M4, and M5). “pre EM” denotes sampling before both MIA and EM; “EM0” denotes sampling immediately after EM; “EM3” denotes sampling on day 3 after EM; “EM4” denotes sampling on day 4 after EM; “EM5” denotes sampling on day 5 after EM

In the nasopharynx of the four autopsy cases, the virus was detected before and after EM (Tables 2 and 3, Fig. 2A, and Supplementary Files 1 and 2), with viral loads reduced by > 99% compared with that of pre-EM levels. After EM, the viral load decreased in case A2, remained largely unaltered in A3, and increased in A1 and A4. In contrast, although the virus was detected in the oropharynx of all four autopsy cases before EM, it was not detected in A3 and A4 after EM. In the two cases in which the virus persisted, the reduction exceeded 99%. The viral load decreased in A2 after EM but remained almost unchanged in A1. On day 3 after EM, the nasopharynx in A4 exhibited the highest viral load (1,140 copies/µL, Ct = 26.1) (Fig. 2A and Supplementary Files 1 and 2). All anal samples were PCR-negative before and after EM and remained negative throughout the study period (data not shown).

**Table 2:** Detection of samples after EM of cadavers

Sample type	Group	EM timing	Detected/Total (%)
Nasopharynx	Autopsy	≤ 1 day	4/4 (100)
		≥ 3 days	4/4 (100)
	MIA	≤ 1 day	3/5 (60)
		≥ 3 days	2/5 (40)
Oropharynx	Autopsy	≤ 1 day	2/4 (50)
		≥ 3 days	2/4 (50)
	MIA	≤ 1 day	1/5 (20)
		≥ 3 days	1/5 (20)
External body surfaces	Autopsy	≤ 1 day	6/17 (35.3)
		≥ 3 days	7/17 (41.2)
	MIA	≤ 1 day	1/9 (11.1)
		≥ 3 days	4/9 (44.4)

This table shows the number of cases in which the virus was detected in the nasopharynx, oropharynx, and on external body surfaces. Sampling after EM was performed on the day after EM, at 3–4 d, and at 7 d for autopsy specimens (*n* = 4), and immediately after EM, at 3 d, and at 4–5 d for MIA specimens (*n* = 5)

**Table 3:** Ct values and viral loads in pharynx before and after EM in autopsy and MIA cases

Sample type	Group	Time point	Ct value (median), (range)	Viral load (median), (range)
Nasopharynx	Autopsy	Pre-EM	22.8 (18.7–24.2)	1.5 × 10⁶ (3.2 × 10^5^–4.3 × 10⁶)
		Day 1 after EM	33.3 (31.3–34.8)	3.1 × 10^1^ (2.1–3.6 × 10^2^)
		Day 3–4 after EM	29.2 (26.1–34.6)	1.0 × 10^3^ (1.9 × 10^1^–1.2 × 10^4^)
		Day 7 after EM†	31.5 (28.4–39.1)	1.3 × 10^2^ (2.2–3.4 × 10^2^)
	MIA	Pre-EM	18.9 (15.2–27.1)	4.99 × 10^5^ (7.6 × 10^2^–2.04 × 10⁶)
		Immediately after EM	35.7 (23.1–40)	0.9 (0–5.9 × 10^3^)
		Day 3–5 after EM	40 (28.2–40)	0 (0–1.6 × 10^2^)
Oropharynx	Autopsy	Pre-EM	25.7 (20.4–28.8)	1.7 × 10^5^ (1.2 × 10^3^–1.3 × 10⁶)
		Day 1 after EM	36.7 (32.1–40)	3.0 (0–118)
		Day 3–4 after EM	39.2 (31.5–40)	0.65 (0–15.3)
		Day 7 after EM†	40 (32.1–40)	0 (0–10.5)
	MIA	Pre-EM	23.8 (19.2–31.1)	2.22 × 10^4^ (5.1 × 10^1^–1.51 × 10^5^)
		Immediately after EM	40 (33.6–40)	0 (0–3.9)
		Day 3–5 after EM	40 (36.6–40)	0 (0–0.5)

Nasopharyngeal and oropharyngeal samples were collected before EM and at defined time points after EM in autopsy (*n* = 4) and MIA (*n* = 5) cases. In autopsy cases, samples were obtained on the day, at 3–4 d, and at 7 d after EM; samples collected at 7 d were available in three cases, whereas samples at the other time points were obtained in four cases. In MIA cases, samples were obtained immediately after EM and at 3–5 d after EM. Data are presented as median (range). Viral loads are shown as copies/µL

### Real-time PCR results for 17 external sites after EM in autopsy cases

**Fig. 3:**
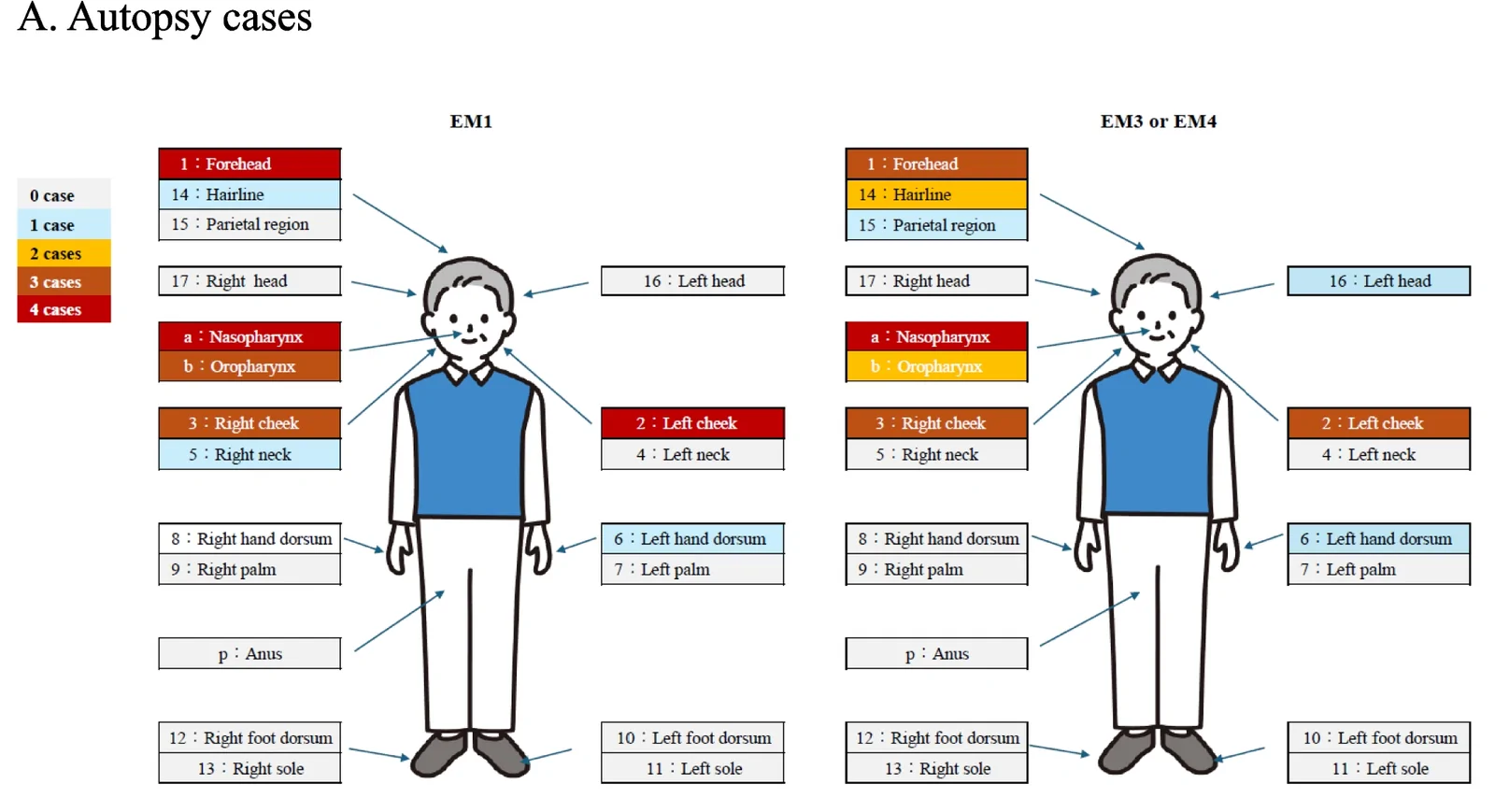

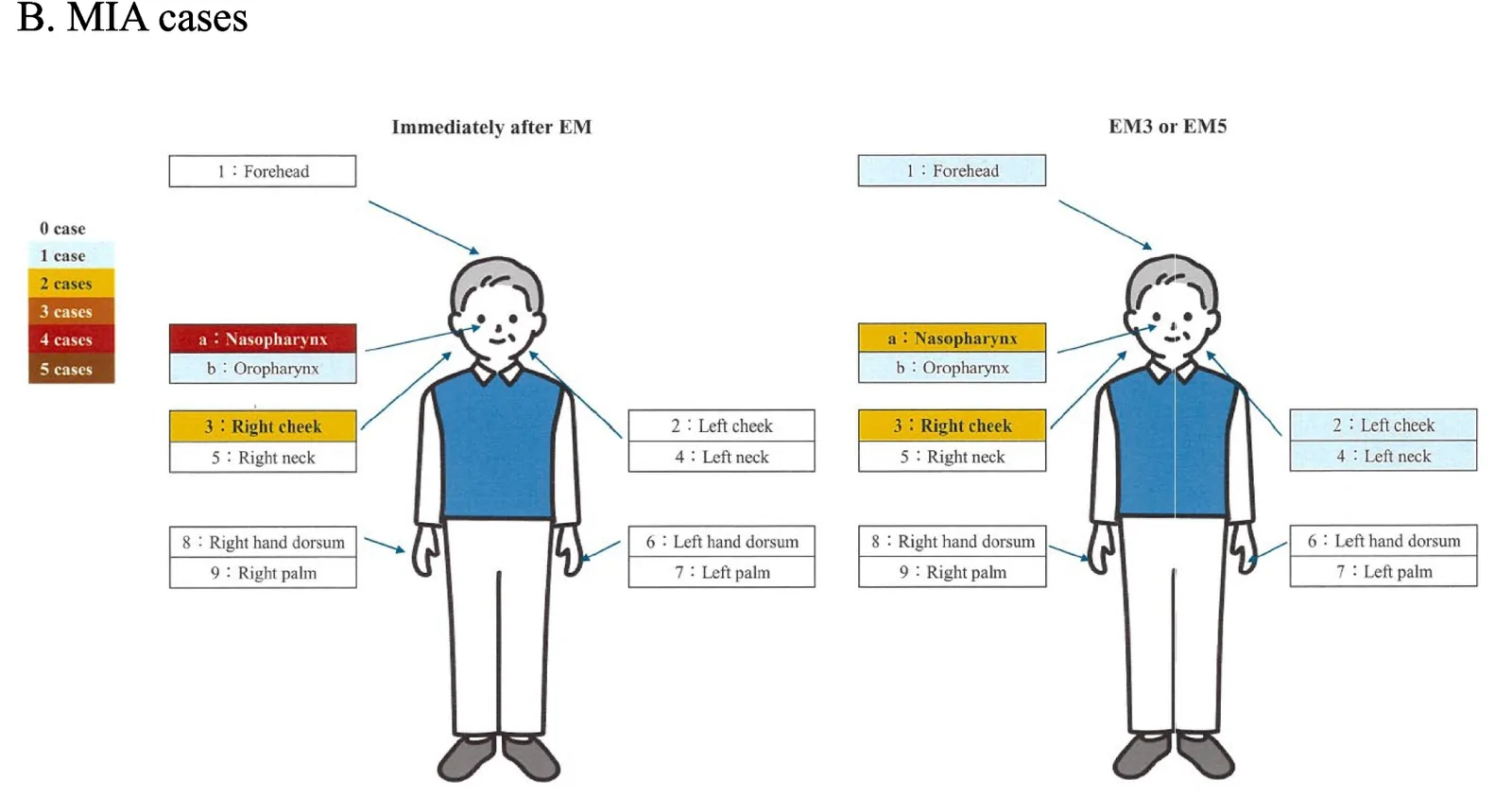
SARS-CoV-2 detection on external body surfaces after EM of cadavers infected with COVID-19. **A** Autopsy cases. The number of virus detections at 17 external sites on the day after and 3 or 4 d after EM implementation in four autopsy cases is shown. Red indicates detection in all four cases, orange in three cases, yellow in two cases, light blue in one case, and light gray indicates no detection. **B** MIA cases. The number of virus detections at nine external sites immediately after EM implementation and 3 or 5 d later in five MIA cases is shown. Brown indicates all five cases, red indicates four cases, orange indicates three cases, yellow indicates two cases, light blue indicates one case, and light gray indicates no detection

The virus was detected at six of 17 external sites (35%) 1 d after EM and at seven sites (41%) ≥ 3 d after EM (Table 2, Fig. 3A and Supplementary File 2).

The virus was consistently detected on the forehead and left cheek in all cases 1 d after EM, and in three cases (A1, A2, and A4), ≥ 3 d after EM. On the right cheek, the virus was detected in three cases (A1, A3, and A4), regardless of the interval after EM. In the hairline region, the virus was detected in A2 1 d after EM and in A1 and A3 ≥ 3 d after EM.

Among all post-EM virus-positive sites, the vertex in A1 exhibited the highest viral load (38.9 copies/µL), with a Ct value of 30.2 on day 4 (Supplementary File 2).

### Real-time PCR results for nasopharynx and oropharynx before and after EM in MIA cases

In all five cases, the virus was detected in the nasopharynx and oropharynx before EM. After EM, the virus was not detected in the nasopharynx of M1 and M2 but was detected in M3, M4, and M5 immediately after EM and in M3 and M5 ≥ 3 d after EM (Table 2 and 3, Fig. 2B, and Supplementary Files 3 and 4). In all cases, the viral load before and after EM was reduced by > 99%. The viral load varied based on the post-EM course: it increased in M3, decreased in the N2 region of M4, and in M5, increased in the N1 region, but decreased in the N2 region.

In the oropharynx, the virus was detected in M3 immediately after EM and in M2 3 d after EM. In both cases, the viral load before and after EM was reduced by > 99%. The viral load increased in M2 and decreased in M3 after EM.

Among all post-EM virus-positive sites, the nasopharynx in M5 exhibited the highest viral load (5,907 copies/µL), with a Ct value of 23.1 (Supplementary Files 3 and 4).

### Real-time PCR results for nine external sites before and after EM in MIA cases

The virus was detected at one site (11%) immediately after EM and four sites (44%) ≥ 3 d post-EM (Table 2 and Fig. 3B). The virus was detected in M4 and M5 in the right cheek immediately after EM and 3 d post-EM. In M5, 5 d after EM, the virus was detected in the forehead, left cheek, and left neck. Among all post-EM virus-positive sites, the left cheek on M5 exhibited the highest viral load (8.89 copies/µL), with a Ct value of 32.4 on day 5 (Supplementary File 4).

In PCR-positive post-EM samples, the viral load before and after EM was reduced by 94%.

### Statistical analysis of virus detection in autopsy and MIA cases

A two-tailed Fisher's exact test revealed no significant differences between the autopsy and MIA groups at any time point.

## Discussion

In the nine COVID-19 cases examined by autopsy or MIA, viral antigens were no longer detected in the nasopharynx after EM, and viral loads were reduced by > 99% in the nasopharynx and oropharynx immediately after EM. The antigen test is an immunochromatographic assay that detects viral proteins (antigens) through antigen–antibody reactions. In samples treated with formaldehyde after EM, antigenicity appears to be blocked because formaldehyde cross-links proteins and peptides, thereby fixing the antigens and preventing detection. Across all post-EM PCR-positive samples, the viral load was reduced by 99% in the nasopharynx and oropharynx and by 94% on external body surfaces compared with that of pre-EM levels. Although there were positive PCR detections, the viral load was extremely low. Similarly, Vymazalová et al. [9] reported that fixative treatment significantly reduces SARS-CoV-2 RNA detection in cadavers. Together, these data indicate that, after autopsy or MIA, EM-based preservation and disinfection significantly reduce the viral load in the nasopharynx, oropharynx, and outer surfaces of cadavers with COVID-19.

The virus was detected in the nasopharynx across all cases (100%) both 1 d and ≥ 3 d after autopsy. Following MIA, the detection rate was 60% (three cases) immediately after EM and 40% (two cases) ≥ 3 d (Table 2 and Fig. 2). In the oropharynx, the detection rates remained at 50% in autopsy cases and 20% in MIA cases at both time points (Table 2 and Fig. 2). Therefore, EM was more effective in autopsy than in MIA cases. Statistical analysis revealed no significant differences, though this may be an artifact of the small sample size.

In conventional EM, the drug solution is injected into an artery, perfused throughout the body, and drained through a vein, allowing intravascular pressure to distribute the fixative to the capillaries and tissues, while dehydration occurs via osmotic effects and formaldehyde action. However, in autopsy cases, major vessels such as the aorta and veins are often severed or removed, preventing the maintenance of intravascular pressure and impairing whole-body perfusion. Consequently, treatment in autopsy cases relies more heavily on wiping, packing, and subcutaneous injection, particularly in the head and torso. In contrast, MIA preserves vascular continuity, allowing perfusion similar to that in non-autopsied bodies. These procedural differences likely explain the generally lower detection rates in MIA cases.

During the EM procedures for all COVID-19-induced deaths, the nasal cavity, nasopharynx, oral cavity, and oropharynx were wiped with cotton swabs soaked in formaldehyde or coated with a gel-like pack. However, considering that the virus persisted in all autopsy cases (100%) on both the day after EM and 3 d later, simply wiping the nasal cavity with a chemical solution proved insufficient. During autopsy, the arteries above the internal and external carotid arteries may be severed, suggesting that the perfusion of the chemical solution to the head and face depends on the remaining arteries. If all the arteries around the neck were intact, the solution should be sufficiently perfused to the head/facial tissues, but the location of the cut site may have had an effect. This would explain the consistent detection of the virus in the nasopharynx, even though EM was performed immediately after dissection. Furthermore, in four of the nine cases, the viral load increased in the nasopharynx and, in two cases, it increased in the oropharynx 3 d after EM. In 2024, Nagasawa et al. reported that the infectious virus levels in the nasopharynx of a patient who died during the viral shedding phase did not decrease even 12 d after death [23]. This suggests insufficient penetration of the EM solution, and that appropriate infection control measures are necessary even for bodies that have undergone EM treatment.

Viral testing of anal swabs was performed on autopsied cadavers before and after EM, but the results were negative in both cases. COVID-19 can cause gastrointestinal symptoms, such as diarrhea, abdominal pain, nausea, and vomiting. The virus can be detected in the feces and anus. Anal swabs have been used as a diagnostic tool for COVID-19 [24, 25]; however, these four cases were not considered. Therefore, although this study did not assess the effectiveness of EM on the anus, the findings indicated a low risk of viral transmission from the head, neck, or lungs to the anus during autopsy and EM procedures.

Regarding external detection, the virus was detected in 6/17 (35%) sites on the day after and in 7/17 (41%) sites ≥ 3 d after EM, demonstrating minimal alteration in the detection rate over time (Table 2 and Fig. 3A). Among the nine external sites examined for the MIA cases, the virus was detected at one site (11%) immediately after EM, increasing to four sites (44%) ≥ 3 d after EM (Table 2 and Fig. 3B). In this study, the bodies after EM were stored in a refrigerator using new disposable gowns and body bags, and specimen collection was performed on a laminar flow dissection table with specimen collectors wearing PPE, inhibiting external contamination. Our results indicate that attention should be paid to external infection on the upper body. Furthermore, while it was necessary to thoroughly cleanse blood and other substances adhering to the body surface of autopsy specimens and ensure disinfection, no difference was observed in the number of sites where viruses were detected between the autopsy and MIA cases. Consequently, the number of detected sites indicates that thorough disinfection of external sites is crucial, regardless of whether the body has undergone autopsy or MIA.

This study demonstrated that EM reduces the detection rate of SARS-CoV-2 in cadavers with a history of COVID-19 and significantly decreases the viral load. This effectively lowers the risk of infection during post-mortem handling. The virus detection rates were lower in MIA cases than in autopsied cases, suggesting that combining MIA with EM may improve biosafety. These findings support the continuation of medical education involving cadavers, including autopsy and dissection training, and funeral-related operations during infectious disease outbreaks. However, since viral particles may persist in the nasopharynx and on body surfaces, continued adherence to standard precautions and appropriate EM and disinfection, particularly in the head and neck region, remain crucial even after EM treatment. Considering the potential for emerging and re-emerging infectious diseases, our findings reaffirm the significance of identifying infection routes and implementing safety protocols when handling cadavers with infectious diseases in postmortem diagnosis, medical education, and funeral preparations.

### Limitations

Autopsies and MIA were performed with the consent of the bereaved families of the patients who died of COVID-19 and were embalmed. However, owing to the challenges in contacting bereaved families and obtaining consent, the sample size was small. For similar reasons, we could not use cases from the control group. Additionally, although the statistical analysis revealed no significant differences, the small sample size may have resulted in insufficient statistical power.

We assessed viral genes on the exterior surfaces of corpses with COVID-19; however, cell culture was not performed on any sample. SARS-CoV-2 is classified as a specific pathogen under the Infectious Diseases Control Act, and procedures that could not be conducted in our laboratory, such as isolation, cultivation, and identification, should be conducted in a Biosafety Level 3 facility.

The reagents and methods used for nucleic acid extraction and real-time PCR differed between pre-autopsy (August 2021 to February 2022) and MIA specimens and post-EM real-time PCR tests (after April 2022). However, because clinical specimens were prioritized for real-time PCR, it was challenging to use standardized reagents.

Therefore, the results of this study are exploratory in nature rather than confirmatory. Nevertheless, assessing viral levels in nasopharyngeal, oropharyngeal, and anal swab samples— including 17 and 9 samples from corpses following autopsy and MIA, respectively—while considering the time elapsed after EM is important for assessing the infection risk associated with cadavers.

## Conclusion

Antigen testing of the nasopharynx after EM in cadavers with COVID-19 yielded negative results. In real-time PCR tests, SARS-CoV-2 was detected in the nasopharynx, oropharynx, and external surface areas of cadavers, even after EM in certain specimens. However, the detection rate was reduced by > 99% in the nasopharynx and oropharynx in all cases, and > 94% on the external body surfaces in MIA cases. Virus detection rates in the nasopharynx and oropharynx were lower in cadavers following MIA than autopsy, highlighting the significance of disinfection and sterilization of external surfaces. Although viral load and infectivity do not always correlate perfectly, a decrease in the viral load generally lowers the risk of infection. Therefore, our results suggest that performing MIA for postmortem diagnosis followed by disinfection using EM is effective in reducing the risk of SARS-CoV-2 infection among personnel involved in postmortem examinations and cadaver handling.

## Supplementary Information

Below is the link to the electronic supplementary material.

Supplementary file1 (PDF 124 KB)

Supplementary file2 (PDF 172 KB)

Supplementary file3 (PDF 118 KB)

Supplementary file4 (PDF 202 KB)

## Data Availability

The data are not publicly available due to ethical and privacy restrictions but are available from the corresponding author upon reasonable request.
