# Supplementary material for: Assessing the effectiveness of embalming in cadavers with COVID-19 after full autopsy and minimally invasive autopsy
Source: Forensic Sci Med Pathol. 2026 Apr 10;22(3):866–76. doi: 10.1007/s12024-026-01225-6 (PMC13672653; doi:10.1007/s12024-026-01225-6)
Supplement: Supplementary file 1 [file 12024_2026_1225_MOESM1_ESM.pdf]

## Supplementary file 1 Ct values, viral loads, and reduction rates before and after EM for SARS-CoV-2 (N2 region) in autopsy cases

**A. Nasopharynx**

| Case number | Pre-EM | | The next day after EM | | | 3 or 4 days after EM | | | 7 days after EM | | |
|---|---|---|---|---|---|---|---|---|---|---|---|
| | Ct values | Viral loads (copies/μL) | Ct values | Viral loads (copies/μL) | Reduction rates (%) | Ct values | Viral loads (copies/μL) | Reduction rates (%) | Ct values | Viral loads (copies/μL) | Reduction rates (%) |
| **A1** | 22.2 | 4,274,000 | 34.8 | 2.11 | 100 | 28.5 | 12,400 | 99.71 | 28.4 | 131 | 99.97 |
| **A2** | 24.2 | 367,500 | 34.4 | 24.9 | 99.99 | 34.6 | 18.7 | 99.99 | 39.1 | 2.17 | 99.99 |
| **A3** | 23.4 | 2,705,500 | 32.2 | 355 | 99.99 | 29.9 | 864 | 99.97 | 31.5 | 344 | 99.99 |
| **A4** | 18.7 | 324,600 | 31.3 | 36.7 | 99.99 | 26.1 | 1,140 | 99.65 | | not tested | |

**B. Oropharynx**

| Case number | Pre-EM | | The next day after EM | | | 3 or 4 days after EM | | | 7 days after EM | | |
|---|---|---|---|---|---|---|---|---|---|---|---|
| | Ct values | Viral loads (copies/μL) | Ct values | Viral loads (copies/μL) | Reduction rates (%) | Ct values | Viral loads (copies/μL) | Reduction rates (%) | Ct values | Viral loads (copies/μL) | Reduction rates (%) |
| **A1** | 28.8 | 1,197 | 33.3 | 6.08 | 99.49 | 31.5 | 15.3 | 98.72 | 32.1 | 10.5 | 99.12 |
| **A2** | 23.2 | 1,284,500 | 32.1 | 118 | 99.99 | 38.4 | 1.3 | 99.99 | 40 | 0 | 100 |
| **A3** | 28.1 | 252,350 | 40 | 0 | 100 | 40 | 0 | 100 | 40 | 0 | 100 |
| **A4** | 20.4 | 97515 | 40 | 0 | 100 | 40 | 0 | 100 | | not tested | |
