# Supplementary material for: Assessing the effectiveness of embalming in cadavers with COVID-19 after full autopsy and minimally invasive autopsy
Source: Forensic Sci Med Pathol. 2026 Apr 10;22(3):866–76. doi: 10.1007/s12024-026-01225-6 (PMC13672653; doi:10.1007/s12024-026-01225-6)
Supplement: Supplementary file 2 [file 12024_2026_1225_MOESM2_ESM.pdf]

Supplementary File 2 Ct values and viral loads after EM for SARS-CoV-2 (N1 and N2 regions) in autopsy cases

Case number	Regions of SARS-CoV-2	Date after EM	Measurement value	a<br>Nasopharynx	b<br>Oropharynx	p<br>Anus	1<br>Forehead	2<br>Left cheek	3<br>Light cheek	4<br>Left neck	5<br>Right neck	6<br>Left hand dorsum	7<br>Left palm	8<br>Right hand dorsum	9<br>Right palm	10<br>Left foot dorsum	11<br>Left sole	12<br>Right foot dorsum	13<br>Right sole	14<br>Hairline	15<br>Parietal region	16<br>Left head	17<br>Right head
A1	N1	EM1	Ct values	37.0	35.3	-	37.7	-	-	-	-	-	-	-	-	-	-	-	-	-	-	-	-
			Quantitative value/1μL	1.24E+00	4.65E+00	-	1.03E+00	-	-	-	-	-	-	-	-	-	-	-	-	-	-	-	-
		EM4	Ct values	32.2	34.5	-	-	38.4	38.9	-	-	-	-	-	-	-	-	-	-	38.7	33.5	37.5	-
			Quantitative value/1μL	3.16E+01	6.19E+00	-	-	4.20E-01	2.90E-01	-	-	-	-	-	-	-	-	-	-	3.30E-01	1.31E+01	8.40E-01	-
	N2	EM1	Ct values	34.8	33.3	-	33.9	34.6	37.1	-	-	-	-	-	-	-	-	-	-	-	-	-	-
			Quantitative value/1μL	2.11E+00	6.08E+00	-	3.96E+00	2.38E+00	4.01E-01	-	-	-	-	-	-	-	-	-	-	-	-	-	-
		EM4	Ct values	28.5	31.5	-	36.9	36.0	35.1	-	-	-	-	-	-	-	-	-	-	37.1	30.2	35.4	-
			Quantitative value/1μL	1.24E+02	1.53E+01	-	3.20E-01	6.20E-01	.1.16	-	-	-	-	-	-	-	-	-	-	2.90E-01	3.89E+01	9.40E-01	-
A2	N1	EM1	Ct values	36.2	33.1	-	38.1	39.9	40.8	40.0	-	-	-	-	-	-	-	-	-	39.4	-	-	40.0
			Quantitative value/1μL	1.71E+01	7.11E+01	-	2.20E+00	6.00E-01	3.00E-01	5.00E-01	-	-	-	-	-	-	-	-	-	9.00E-01	-	-	6.00E-01
		EM3	Ct values	35.9	39.6	-	39.6	40.0	-	-	-	-	-	-	-	-	-	-	40.3	-	-	-	-
			Quantitative value/1μL	1.24E+01	9.00E-01	-	9.00E-01	7.00E-01	-	-	-	-	-	-	-	-	-	-	5.00E-01	-	-	-	-
	N2	EM1	Ct values	34.4	32.1	-	37.0	39.9	40.0	40.1	40.0	-	-	-	-	-	-	-	-	39.5	-	-	-
			Quantitative value/1μL	2.49E+01	1.18E+02	-	4.00E+00	5.00E-01	5.00E-01	5.00E-01	5.00E-01	-	-	-	-	-	-	-	-	7.00E-01	-	-	-
		EM3	Ct values	34.6	38.4	-	38.9	40.9	-	-	-	-	-	-	-	-	-	40.1	40.5	-	-	-	-
			Quantitative value/1μL	1.87E+01	1.30E+00	-	1.28E+00	2.20E-01	-	-	-	-	-	-	-	-	-	3.90E-01	2.90E-01	-	-	-	-
A3	N1	EM1	Ct values	35.0	40.1	-	-	38.7	-	-	-	39.5	-	-	-	-	-	-	-	-	-	-	-
			Quantitative value/1μL	1.15E+02	4.75E+00	-	-	8.10E-01	-	-	-	5.00E-01	-	-	-	-	-	-	-	-	-	-	-
		EM3	Ct values	31.5	-	-	-	38.0	-	-	-	-	-	-	-	-	-	-	-	-	-	-	-
			Quantitative value/1μL	3.29E+02	-	-	-	1.29E+00	-	-	-	9.50E-01	-	-	-	-	-	-	-	-	-	-	-
	N2	EM1	Ct values	32.2	40.4	-	38.1	38.6	38.8	-	-	39.1	-	-	-	-	-	-	-	39.9	-	-	-
			Quantitative value/1μL	3.55E+02	2.91E+00	-	2.33E+00	1.67E+00	1.71E+00	-	-	1.22E+00	-	-	-	-	-	-	-	6.80E-01	-	-	-
		EM3	Ct values	29.9	-	-	-	-	37.1	-	-	-	-	-	-	-	-	-	-	-	-	-	-
			Quantitative value/1μL	8.64E+02	-	-	-	-	6.36E-01	-	-	-	-	-	-	-	-	-	-	-	-	-	-
A4	N1	EM1	Ct values	34.9	-	-	-	39.3	39.5	-	-	-	-	-	-					-	-		
			Quantitative value/1μL	1.29E+01	-	-	-	7.27E-01	6.50E-01	-	-	-	-	-	-					-	-		
		EM3	Ct values	28,6	-	-	-	38.2	37.5	-	-	-	-	-	-					-	-		
			Quantitative value/1μL	5.09E+02	-	-	-	9.60E-01	1.53E+00	-	-	-	-	-	-					-	-		
	N2	EM1	Ct values	31.3	-	-	37.1	35.6	38.6	-	36.8	-	-	-	-					-	-	-	-
			Quantitative value/1μL	3.67E+01	-	-	8.71E-01	2.23E+00	3.19E-01	-	1.05E+00	-	-	-	-					-	-	-	-
		EM3	Ct values	26.1	-	-	36.6	36.9	36.6	-	-	-	-	-	-					-	-	-	-
			Quantitative value/1μL	1.14E+03	-	-	1.38E+00	1.11E+00	1.36E+00	-	-	-	-	-	-					-	-	-	-
