# Supplementary material for: Assessing the effectiveness of embalming in cadavers with COVID-19 after full autopsy and minimally invasive autopsy
Source: Forensic Sci Med Pathol. 2026 Apr 10;22(3):866–76. doi: 10.1007/s12024-026-01225-6 (PMC13672653; doi:10.1007/s12024-026-01225-6)
Supplement: Supplementary file 3 [file 12024_2026_1225_MOESM3_ESM.pdf]

Supplementary file 3　Ct values, viral loads, and reduction rates before and after EM for SARS-CoV-2 (N2 region) in MIA cases

A. Nasopharynx

Case number	Pre-EM		Immediately after EM			3, 4 or 5 days after EM		
	Ct values	Viral loads（copies/μL）	Ct values	Viral loads（copies/μL）	Reduction rates（%）	Ct values	Viral loads（copies/μL）	Reduction rates（%）
M1	27.1	763	40	0	100	40	0	100
M2	15.2	2,043,121	40	0	100	40	0	100
M3	21.6	49,884	35.7	0.90	99.99	33.4	4.58	99.99
M4	18.9	498,906	34.1	2.83	99.99	40	0	100
M5	17.9	852,232	23.1	5907	99.31	28.2	162	99.98

B. Oropharynx

Case number	Pre-EM		Immediately after EM			3, 4 or 5 days after EM		
	Ct values	Viral loads（copies/μL）	Ct values	Viral loads（copies/μL）	Reduction rates（%）	Ct values	Viral loads（copies/μL）	Reduction rates（%）
M1	31.1	51.2	40	0	100	40	0	100
M2	19.2	149,346	40	0	100	36.6	0.50	99.99
M3	24.1	10,209	33.6	3.88	99.96	40	0	100
M4	23.8	22,154	40	0	100	40	0	100
M5	20.1	151,360	40	0	100	40	0	100
