# Supplementary material for: Assessing the effectiveness of embalming in cadavers with COVID-19 after full autopsy and minimally invasive autopsy
Source: Forensic Sci Med Pathol. 2026 Apr 10;22(3):866–76. doi: 10.1007/s12024-026-01225-6 (PMC13672653; doi:10.1007/s12024-026-01225-6)
Supplement: Supplementary file 4 [file 12024_2026_1225_MOESM4_ESM.pdf]

## Supplementary File 4　　Ct values and viral loads before and after EM for SARS-CoV-2 (N1 and N2 regions) in MIA cases

| Case number | Regions of SARS-CoV-2 | Time elapsed before or after EM | Measurement value | A | | B | | 1 | | 2 | | 3 | | 4 | | 5 | 6 | 7 | 8 | 9 |
|---|---|---|---|---|---|---|---|---|---|---|---|---|---|---|---|---|---|---|---|---|
| | | | | Nasopharynx | Reduction rate | Oropharynx | Reduction rate | Forehead | Reduction rate | Left cheek | Reduction rate | Right cheek | Reduction rate | Left neck | Reduction rate | Right neck | Left hand dorsum | Left palm | Right hand dorsum | Right palm |
| M1 | N1 | pre EM | Ct values | 30.87572098 | | 33.74578857 | | | | | | | | | | | | | | |
| | | | Quantitative value/1μL | 207.1434937 | | 28.1879673 | | | | | | | | | | | | | | |
| | | post EM 0 | Ct values | – | | – | | – | | – | | – | | – | | – | – | – | – | – |
| | | | Quantitative value/1μL | – | | – | | – | | – | | – | | – | | – | – | – | – | – |
| | | post EM 3 | Ct values | – | | – | | – | | – | | – | | – | | – | – | – | – | – |
| | | | Quantitative value/1μL | – | | – | | – | | – | | – | | – | | – | – | – | – | – |
| | N2 | pre EM | Ct values | 27.10246086 | | 31.14392281 | | | | | | | | | | | | | | |
| | | | Quantitative value/1μL | 763.0012207 | | 51.16779327 | | | | | | | | | | | | | | |
| | | post EM 0 | Ct values | – | | – | | – | | – | | – | | – | | – | – | – | – | – |
| | | | Quantitative value/1μL | – | | – | | – | | – | | – | | – | | – | – | – | – | – |
| | | post EM 3 | Ct values | – | | – | | – | | – | | – | | – | | – | – | – | – | |
| | | | Quantitative value/1μL | – | | – | | – | | – | | – | | – | | – | – | – | – | |
| M2 | N1 | pre EM | Ct values | 18.92073059 | | 23.62431335 | | 33.07967758 | | 29.70692825 | | 25.27260685 | | 37.39520073 | | 34.84254837 | 36.72973633 | 37.32209396 | 35.89205933 | 28.43309021 |
| | | | Quantitative value/1μL | 568758.375 | | 24492.69922 | | 43.98425293 | | 430.952301 | | 8141.213135 | | 2.5802401 | | 13.55527973 | 3.837823868 | 2.578021049 | 7.071504593 | 95989.98438 |
| | | post EM 0 | Ct values | – | | – | | – | | – | | – | | – | | – | – | – | – | – |
| | | | Quantitative value/1μL | – | | – | | – | | – | | – | | – | | – | – | – | – | – |
| | | post EM 5 | Ct values | – | | – | | – | | – | | – | | – | | – | – | – | – | – |
| | | | Quantitative value/1μL | – | | – | | – | | – | | – | | – | | – | – | – | – | – |
| | N2 | pre EM | Ct values | 15.24453163 | | 19.1675148 | | 29.7065773 | | 26.40912056 | | 21.61328506 | | 33.64771271 | | 31.73421288 | 33.82888031 | 36.02285385 | 33.64160156 | 32.96166992 |
| | | | Quantitative value/1μL | 2043121.75 | | 149346.4375 | | 132.4781799 | | 1194.18335 | | 29240.46484 | | 10.43440056 | | 34.28157806 | 8.759128571 | 2.497719765 | 9.644962311 | 15.81963921 |
| | | post EM 0 | Ct values | – | | – | | – | | – | | – | | – | | – | – | – | – | – |
| | | | Quantitative value/1μL | – | | – | | – | | – | | – | | – | | – | – | – | – | – |
| | | post EM 5 | Ct values | – | | 36.55137253 | | – | | – | | – | | – | | – | – | – | – | – |
| | | | Quantitative value/1μL | – | | 0.498100728 | **99.9997%** | – | | – | | – | | – | | – | – | – | – | – |
| M3 | N1 | pre EM | Ct values | 23.97686768 | | 26.55195618 | | 34.70641327 | | 34.02233887 | | 31.70303726 | | – | | 37.08005524 | 38.0376358 | 33.73384476 | – | 31.13578987 |
| | | | Quantitative value/1μL | 12528.67969 | | 2118.222656 | | 7.93217659 | | 12.19819927 | | 61.46862793 | | – | | 1.472954154 | 0.760459065 | 14.95312881 | – | 89.77552795 |
| | | post EM 0 | Ct values | 37.87342834 | | 35.98989868 | | – | | – | | – | | – | | – | – | – | – | – |
| | | | Quantitative value/1μL | 0.379007638 | **99.9970%** | 1.587103844 | **99.9251%** | – | | – | | – | | – | | – | – | – | – | – |
| | | post EM 4 | Ct values | 34.60398102 | | – | | – | | – | | – | | – | | – | – | – | – | – |
| | | | Quantitative value/1μL | 4.080571175 | **99.9674%** | – | | – | | – | | – | | – | | – | – | – | – | – |
| | N2 | pre EM | Ct values | 21.59589767 | | 24.07616234 | | 32.98846436 | | 32.07012558 | | 29.38076782 | | 36.36348343 | | 36.30615997 | 36.00913239 | 31.41131592 | 36.2151413 | 28.94106865 |
| | | | Quantitative value/1μL | 49884.30469 | | 10208.90625 | | 34.3928566 | | 61.73193359 | | 343.8041992 | | 3.969593525 | | 4.521867275 | 5.019868851 | 93.94709778 | 4.46117115 | 456.1470337 |
| | | post EM 0 | Ct values | 35.70040131 | | 33.62631989 | | – | | – | | – | | – | | – | – | – | – | – |
| | | | Quantitative value/1μL | 0.904821634 | **99.9982%** | 3.878847361 | **99.9620%** | – | | – | | – | | – | | – | – | – | – | – |
| | | post EM 4 | Ct values | 33.36380005 | | – | | – | | – | | – | | – | | – | – | – | – | – |
| | | | Quantitative value/1μL | 4.577113152 | **99.9908%** | – | | – | | – | | – | | – | | – | – | – | – | – |
| M4 | N1 | pre EM | Ct values | 22.13912773 | | 27.08109093 | | 32.23148346 | | 30.07852173 | | 32.77284431 | | 33.6717453 | | 36.11522293 | 31.77053452 | 29.40716171 | 32.77519989 | 32.17202377 |
| | | | Quantitative value/1μL | 91885.5 | | 3887.37207 | | 147.2446747 | | 581.4419556 | | 104.3685036 | | 58.83382416 | | 12.42793465 | 196.6964111 | 883.9156494 | 103.6855774 | 155.085144 |
| | | post EM 0 | Ct values | – | | – | | – | | – | | 36.05766678 | | – | | – | – | – | – | – |
| | | | Quantitative value/1μL | – | | – | | – | | – | | 1.493879795 | **98.5686%** | – | | – | – | – | – | – |
| | | post EM 3 | Ct values | – | | – | | – | | – | | 35.61009979 | | – | | – | – | – | – | – |
| | | | Quantitative value/1μL | – | | – | | – | | – | | 1.983535647 | **98.0995%** | – | | – | – | – | – | – |
| | N2 | pre EM | Ct values | 18.9275322 | | 23.81699753 | | 29.35339928 | | 26.93193054 | | 29.65765381 | | 30.76098633 | | 33.3118782 | 28.47529984 | 26.34010696 | 29.49961472 | 28.69616318 |
| | | | Quantitative value/1μL | 498906.75 | | 22154.73242 | | 647.6695557 | | 3030.4729 | | 532.911499 | | 263.7487793 | | 51.85946655 | 1135.508545 | 4554.838867 | 589.4759521 | 983.769165 |
| | | post EM 0 | Ct values | 34.05361176 | | – | | – | | – | | 31.58407974 | | – | | – | – | – | – | – |
| | | | Quantitative value/1μL | 2.834300995 | **99.9994%** | – | | – | | – | | 15.70116901 | **97.0537%** | – | | – | – | – | – | – |
| | | post EM 3 | Ct values | – | | – | | – | | – | | 33.04507446 | | – | | – | – | – | – | – |
| | | | Quantitative value/1μL | – | | – | | – | | – | | 5.722193718 | **98.9262%** | – | | – | – | – | – | – |
| M5 | N1 | pre EM | Ct values | 19.93848801 | | 22.41312408 | | 30.24368286 | | 32.3923645 | | 26.16266155 | | 38.09039688 | | 37.52616119 | 28.64649963 | 36.50899124 | 36.45293427 | 37.1152916 |
| | | | Quantitative value/1μL | 197455.5469 | | 37708.85547 | | 193.4798889 | | 43.27500916 | | 2969.024048 | | 0.917291045 | | 1.371510863 | 26674.78125 | 2.698043346 | 2.784446716 | 1.773397207 |
| | | post EM 0 | Ct values | – | | – | | – | | – | | – | | – | | – | – | – | – | – |
| | | | Quantitative value/1μL | – | | – | | – | | – | | – | | – | | – | – | – | – | – |
| | | post EM 5 | Ct values | 32.01240921 | | – | | 38.98241043 | | 35.29393005 | | 36.9739151 | | 37.80250549 | | – | – | – | – | – |
| | | | Quantitative value/1μL | 27.52695847 | **99.9861%** | – | | 0.167139262 | **99.9136%** | 2.976305485 | **93.1223%** | 0.724248171 | **99.9756%** | 0.395527273 | **56.8809%** | – | – | – | – | – |
| | N2 | pre EM | Ct values | 17.78550339 | | 20.09040833 | | 28.70829773 | | 30.23548889 | | 23.32829857 | | 36.80024719 | | 34.91180038 | 33.77915955 | 34.23399353 | 34.31369781 | 34.79727173 |
| | | | Quantitative value/1μL | 852232.25 | | 151360.6875 | | 236.8220062 | | 75.31703186 | | 13339.25586 | | 0.553330183 | | 2.255659819 | 5.295635223 | 3.78382206 | 3.642651558 | 2.454254389 |
| | | post EM 0 | Ct values | 23.05179787 | | – | | – | | – | | 37.00094604 | | – | | – | – | – | – | – |
| | | | Quantitative value/1μL | 5906.699219 | **99.3069%** | – | | – | | – | | 0.364510089 | **99.9973%** | – | | – | – | – | – | – |
| | | post EM 5 | Ct values | 28.22859573 | | – | | 33.86821747 | | 32.4040451 | | 35.11841583 | | – | | – | – | – | – | – |
| | | | Quantitative value/1μL | 162.2519226 | **99.9810%** | – | | 3.301763058 | **98.6058%** | 8.893130302 | **88.1924%** | 1.520716906 | **99.9886%** | – | | – | – | – | – | – |

※*The "reduction rate" indicates the ratio of the reduced “virus amount after EM” to the “virus amount at pre EM.”
